# Interface astrogliosis in contact sport head impacts and military blast exposure

**DOI:** 10.1186/s40478-022-01358-z

**Published:** 2022-04-13

**Authors:** Katharine J. Babcock, Bobak Abdolmohammadi, Patrick T. Kiernan, Ian Mahar, Jonathan D. Cherry, Victor E. Alvarez, Lee E. Goldstein, Thor D. Stein, Ann C. McKee, Bertrand R. Huber

**Affiliations:** 1 Department of Anatomy & Neurobiology, Boston University School of Medicine, Boston, MA 02118 USA; 2 VA Boston Healthcare System, 150 S. Huntington Avenue, Boston, MA 02130 USA; 3 Boston University Alzheimer’s Disease and CTE Centers, Boston University School of Medicine, Boston, MA 02118 USA; 4 Department of Pathology and Laboratory Medicine, Boston University School of Medicine, Boston, MA 02118 USA; 5 Department of Neurology, Boston University School of Medicine, Boston, MA 02118 USA; 6 National Center for PTSD, VA Boston Healthcare System, Boston, MA 02130 USA; 7 VA Bedford Healthcare System, Bedford, MA 01730 USA; 8 Molecular Aging and Development Laboratory, Boston University School of Medicine, Boston, MA 02118 USA; 9 Boston University College of Engineering, Boston, MA 02115 USA

**Keywords:** Mild traumatic brain injury, Repetitive head impacts, Blast injury, Chronic traumatic encephalopathy, Astrogliosis

## Abstract

Exposure to military blast and repetitive head impacts (RHI) in contact sports is associated with increased risk of long-term neurobehavioral sequelae and cognitive deficits, and the neurodegenerative disease chronic traumatic encephalopathy (CTE). At present, the exact pathogenic mechanisms of RHI and CTE are unknown, and no targeted therapies are available. Astrocytes have recently emerged as key mediators of the multicellular response to head trauma. Here, we investigated interface astrogliosis in blast and impact neurotrauma, specifically in the context of RHI and early stage CTE. We compared postmortem brain tissue from former military veterans with a history of blast exposure with and without a neuropathological diagnosis of CTE, former American football players with a history of RHI with and without a neuropathological diagnosis of CTE, and control donors without a history of blast, RHI exposure or CTE diagnosis. Using quantitative immunofluorescence, we found that astrogliosis was higher at the grey-white matter interface in the dorsolateral frontal cortex, with mixed effects at the subpial surface and underlying cortex, in both blast and RHI donors with and without CTE, compared to controls. These results indicate that certain astrocytic alterations are associated with both impact and blast neurotrauma, and that different astroglial responses take place in distinct brain regions.

## Introduction

Mild traumatic brain injuries (mTBI), including concussive and subconcussive injuries, are the most common type of TBI, accounting for an estimated 80% of all TBIs [9]. Blast-associated TBI is prevalent in military personnel deployed to Iraq and Afghanistan due to the widespread use of improvised explosive devices (IEDs) and is considered an "invisible" wound of war due to its association with mood and behavioral changes, including post-traumatic stress disorder (PTSD), in the absence of detectable physical damage [18, 25]. Exposure to repetitive head impacts (RHI) in contact sports, a form of mTBI, is also associated with long-term neurobehavioral and cognitive deficits. Both blast and RHI exposure are associated with the development of the neurodegenerative disease chronic traumatic encephalopathy (CTE) [22, 36, 39].

In addition to cognitive and behavior changes, exposure to RHI and military blast is tied to several neuropathological alterations. Athletes with a history of contact sport-related RHI have increased neuroinflammation compared to non-athlete controls, indicated by higher levels of CD68 + macrophages and microglia, that progressively increase in the presence of mild and severe CTE [12]. Other human postmortem studies have identified blood–brain barrier (BBB) alterations with loss of endothelial tight junctions and extravasation of serum proteins, as well as axonal injury, in individuals exposed to RHI with autopsy-confirmed CTE [14, 22, 54]. Post-traumatic BBB disruption, neuroinflammation, and axonal injury have all been demonstrated in animal models of repetitive mild impact and blast TBI [22, 27, 29, 34, 40, 43, 45, 54].

A growing body of evidence implicates astrocytes in many of these post-traumatic injury processes. Astrocytes interact with blood vessels to regulate BBB integrity [1, 4], neuronal synapses to buffer ions and neurotransmitters [6, 44, 46], and oligodendrocytes to regulate myelination [7, 15], and are therefore considered essential in maintaining homeostasis of the brain's microenvironment.

In response to head trauma, astrocytes rapidly undergo structural and functional changes to adopt a reactive phenotype in a process known as astrogliosis [52, 53]. Morphologically, reactive astrocytes are characterized by an upregulation of their main structural protein, glial fibrillary acidic protein (GFAP), resulting in hypertrophy, process enlargement, and increased ramification. Genetic studies have highlighted the heterogeneity of reactive astrocyte responses in various injury settings [57]. Under extreme conditions, astrocytes proliferate and mat together, forming a glial scar that functions as a physical barrier separating injured tissue from surrounding healthy tissue, that can either assist or inhibit repair processes [3, 17, 31, 33, 41]. Non-proliferating reactive astrocytes under less severe injury conditions have also been shown to adopt distinct molecular profiles, with differing functional phenotypes that progress or resolve over time depending on the injury type and severity [16]. For instance, some types of astrocytes release proinflammatory cytokines and neurotoxic molecules in response to CNS infection, while others upregulate homeostatic functions and release neurotrophic factors following stroke or trauma [31, 57].

Astrogliosis is a hallmark neuropathological feature found in virtually all neurodegenerative disorders [53], including CTE [36]. Hsu and colleagues reported increased GFAP + astrogliosis at the grey-white matter junction in the frontal cortex of neuropathologically confirmed late-stage CTE cases, in addition to a degenerative astroglial phenotype characterized by beaded and punctate processes in the underlying white matter [26]. However, no significant differences in astrogliosis or astrocytic degeneration were found in CTE compared to Alzheimer's disease or frontotemporal dementia. Meanwhile, Shivley and colleagues reported a distinctive pattern of astrogliosis at the grey-white matter junction in subjects exposed to military blast, but not in civilians exposed to impact TBI, and concluded that this astroglial pattern was unique to blast injury [50]. However, the Shivley study focused on subjects who experienced single, moderate-to-severe TBI, rather than mild and repetitive injury, and thus did not investigate the effects of RHI frequently experienced in the context of sports.

In the present study, we investigated astrogliosis in brain donors after RHI and RHI-related CTE and compared the results to the severity of the astrocytic alterations after blast and blast-CTE. We hypothesized that astrogliosis would be significantly higher in donors with a history of RHI, regardless of co-existing military blast exposure or CTE diagnosis, compared to age-matched controls without TBI or RHI. We also hypothesized that these effects would be most pronounced in impact-vulnerable regions, such as the grey-white matter interface at the depth of the cortical sulci [20]. We specifically focused on younger brain donors, with low stage CTE, to focus on early disease processes and minimize age-related effects.

## Materials and methods

### Donors

Postmortem human brain tissue was obtained from a convenience sample of 50 donors from 2 brain biorepositories at the VA Boston Healthcare System (summarized in Table 1). Donors were selected if they were between the ages of 20–60, were male, and had brain tissue sections readily available at the time of study. Control tissue was obtained from the National Posttraumatic Stress Disorder (PTSD) brain bank from donors who did not have a military history, TBI exposure, concussion, or any neuropathological diagnosis (n = 7, mean age = 34.6 years ± 2.3). All other brain tissue was obtained from donors (n = 43) belonging to the "Understanding Neurologic Injury and Traumatic Encephalopathy (UNITE)" study at the Veteran's Affairs-Boston University-Concussion Legacy Foundation (VA-BU-CLF) brain bank (UNITE group) [39]. Studies were approved by the institutional review board (IRB) through the Boston University Alzheimer's Disease Center (ADC) and CTE Center, Human Subjects Institutional Review Board of the Boston University School of Medicine, and Edith Nourse Rogers Memorial Veterans Hospital (Bedford, MA).

**Table 1:** Donor Summary Demographics

Cohort	n	Mean Age (yrs)	Mean CTE Stage	Mean Exposure (yrs)
Blast	5	42 ± 3.9	0	0.6 ± 0.40
Blast CTE	5	33 ± 3.5	1.6 ± 0.24	10 ± 2.61
RHI	14	41 ± 2.9	0	10.9 ± 1.10
RHI CTE	19	38 ± 2.8	1.5 ± 0.12	11.2 ± 1.03
Control	7	35 ± 2.3	0	0

Data expressed as ± standard error of the mean (SEM). CTE = Chronic traumatic encephalopathy. RHI = Repetitive head impacts

Brain donors to the UNITE group had a prior history of repetitive mild head trauma through participation in American football, other contact sports, or military blast exposure. Brain tissue cohorts were divided based on their type of neurotrauma exposure and presence or absence of a CTE diagnosis into the following groups: Blast-RHI, Blast-RHI CTE, RHI, and RHI CTE. The Blast-RHI group included individuals with a history of military blast exposure and non-blast related RHI exposure from participation in contact sports (n = 5, mean age = 42 years ± 3.9). The Blast-RHI CTE group included individuals with a history of military blast exposure and non-blast related RHI exposure from contact sports, and a diagnosis of low CTE (n = 5, mean age = 32.8 years ± 3.5, mean CTE stage = 1.6 ± 0.24). The RHI group included individuals with a history of RHI exposure (n = 14, mean age = 41.2 years ± 2.9), all from participation in American football. The RHI CTE group included individuals with a history of RHI exposure from American football and a postmortem diagnosis of low CTE (n = 19, mean age = 37.7 ± 2.8, mean CTE stage = 1.5 ± 0.12).

Due to the small number of brain donors with military blast exposure in the UNITE brain bank, donors with sport histories other than American football were included in the Blast-RHI and Blast-RHI CTE groups. As most donors in the UNITE brain bank have RHI exposure from American football, all donors selected for the RHI and RHI-CTE groups had a history of playing American football.

The neuropathological diagnosis and staging of CTE followed the NINDS/NIBIB criteria for the pathological diagnosis of CTE [5, 35], including the presence of at least one perivascular foci of perivascular p-tau accumulation most commonly found at the depths of the cortical sulci, and the McKee CTE staging scheme [37]. All cases included in this study were free from co-morbid disease, including Alzheimer’s disease (AD), neocortical Lewy Body Disease (LBD), frontotemporal lobar degeneration (FTLD), or motor neuron disease (MND). Additional demographic data for each case are found in Tables 1, 2, 3, 4, 5 and 6.

**Table 2:** Blast-RHI Donor Demographics

Donor	Age	Sex	PMI (hrs)	COD	Blast Exposure	Athletic History	CTE Stage
Blast-RHI 1	33	M	26.5	Other	1	None	0
Blast-RHI 2	36	M	57	Cardiac	11	Youth soccer	0
Blast-RHI 3	40	M	24	Suicide	2	HS FB	0
Blast-RHI 4	46	M	13	Suicide	2	Cycling	0
Blast-RHI 5	55	M	80	Suicide	20	HS FB	0

Data expressed as ± standard error of the mean (SEM). PMI = Post-mortem interval. COD = Cause of death. HS = High school. CTE = Chronic traumatic encephalopathy. RHI = Repetitive head impacts

**Table 3:** Blast-RHI CTE Donor Demographics

Donor	Age	Sex	PMI (hrs)	COD	Blast Exposure	Athletic History	CTE Stage
Blast-RHI CTE 1	22	M	UN	Other	1	HS FB	1
Blast-RHI CTE 2	32	M	19	Accidental	12	MMA	2
Blast-RHI CTE 3	32	M	48	Injury	12	Rugby	2
Blast-RHI CTE 4	34	M	24	Cancer	UN	College FB	1
Blast-RHI CTE 5	44	M	48	Cardiac	1	College FB	2

Data expressed as ± standard error of the mean (SEM). PMI = Post-mortem interval. COD = Cause of death. UN = Unknown. MMA = Mixed martial arts. HS = High school. FB = Football. CTE = Chronic traumatic encephalopathy. RHI = Repetitive head impacts

**Table 4:** RHI Donor Demographics

Donor	Age	Sex	PMI (hrs)	COD	Blast Exposure	Athletic History	CTE Stage
RHI 1	22	M	7	Accidental	0	HS FB	0
RHI 2	22	M	24	Accidental	0	College FB	0
RHI 3	28	M	24	Cardiac	0	College FB	0
RHI 4	38	M	16	Suicide	0	College FB	0
RHI 5	38	M	24	Accidental	0	Pro FB	0
RHI 6	41	M	15	Other	0	HS FB	0
RHI 7	42	M	55	Unknown	0	College FB	0
RHI 8	43	M	48	Suicide	0	HS FB	0
RHI 9	45	M	UN	Suicide	0	HS FB	0
RHI 10	47	M	11	Cancer	0	Football, Hockey	0
RHI 11	49	M	39	Other	0	College FB, Rugby	0
RHI 12	51	M	32	Cardiac	0	Semi-pro FB	0
RHI 13	55	M	38	Cardiac	0	College FB	0
RHI 14	56	M	44	Cardiac	0	College FB	0

Data expressed as ± standard error of the mean (SEM). PMI = Post-mortem interval. COD = Cause of death. UN = Unknown. HS = High school. FB = Football. CTE = Chronic traumatic encephalopathy. RHI = Repetitive head impacts

**Table 5:** RHI CTE Donor Demographics

Donor	Age	Sex	PMI (hrs)	COD	Blast Exposure	Athletic History	CTE Stage
RHI CTE 1	22	M	24	Suicide	0	FB	1
RHI CTE 2	24	M	6	Suicide	0	HS FB	1
RHI CTE 3	24	M	72	Accidental	0	HS FB	1
RHI CTE 4	25	M	22	Suicide	0	HS FB	2
RHI CTE 5	25	M	10	Homicide	0	College FB	1
RHI CTE 6	26	M	29	Injury	0	Pro FB	2
RHI CTE 7	29	M	23	Suicide	0	College FB	2
RHI CTE 8	31	M	19	Cardiac	0	College FB	1
RHI CTE 9	32	M	UN	Suicide	0	College FB	2
RHI CTE 10	40	M	35	Accidental	0	College FB	1
RHI CTE 11	41	M	13	Accidental	0	College FB	2
RHI CTE 12	41	M	24	Suicide	0	College FB	2
RHI CTE 13	45	M	13	Suicide	0	Semi-pro FB, Pro Boxing	2
RHI CTE 14	45	M	24	Suicide	0	FB, motocross	1
RHI CTE 15	46	M	24	Cancer	0	NFL	1
RHI CTE 16	52	M	48	Liver and kidney failure	0	NFL	1
RHI CTE 17	54	M	48	Cardiovascular	0	Pro FB	2
RHI CTE 18	57	M	16	Suicide	0	College FB	1
RHI CTE 19	58	M	48	Cardiovascular	0	HS FB	2

Data expressed as ± standard error of the mean (SEM). PMI = Post-mortem interval. COD = Cause of death. UN = Unknown. HS = High school. FB = Football. NFL = National Football League. CTE = Chronic traumatic encephalopathy. RHI = Repetitive head impacts

**Table 6:** Control Donor Demographics

Donor	Age	Sex	PMI (hrs)	COD	Blast Exposure	Athletic History	CTE Stage
Control 1	22	M	33	Accidental	0	0	0
Control 2	34	M	30	Accidental	0	0	0
Control 3	34	M	32.5	Accidental	0	0	0
Control 4	35	M	38	Suicide	0	0	0
Control 5	37	M	34.5	Accidental	0	0	0
Control 6	40	M	24.5	Cardiac	0	0	0
Control 7	40	M	28	Cardiac	0	0	0

Data expressed as ± standard error of the mean (SEM). PMI = Post-mortem interval. COD = Cause of death. CTE = Chronic traumatic encephalopathy

### Tissue

Tissue processing, neuropathological examination, and tissue storage procedures are harmonized across the UNITE and PTSD brain banks. All brain tissue was comprehensively analyzed neuropathologically to detect the presence of any neurodegenerative disease, according to previously described criteria and protocols [38]. Briefly, postmortem brain tissue was fixed in periodate-lysine-paraformaldehyde (PLP) at 4 °C for at least two weeks before sampling and processing for routine neuropathological workup, which includes paraffin-embedded tissue section staining for hyperphosphorylated tau, amyloid beta, alpha synuclein, transactive response DNA-binding protein 43 (TDP-43), and luxol fast blue- hematoxylin and eosin, as previously described [38, 55]. Samples used in the current study were harvested from the dorsolateral frontal cortex (BA8/9), embedded in paraffin, and sectioned at 20 microns. The dorsolateral frontal cortex was chosen because it is one of the earliest and most affected brain regions in CTE [5].

### Immunofluorescence

Tissue sections underwent antigen retrieval with AR6 (Leica Biosystems; analogous to citrate buffer) for 20 min at 95 degrees Celsius, then blocked for 30 min in 3% donkey serum in phosphate buffered saline with 0.4% triton (PBST). The primary antibody anti-glial fibrillary acidic protein (mouse anti-GFAP, Millipore, 1:750) was applied for one hour, followed by a 30-min incubation with horseradish peroxidase (HRP)-conjugated mouse secondary antibody. Slides were incubated with a fluorescent dye catalyzed by HRP (Opal 520, Perkin Elmer) for 10 min, followed by the nuclear counterstain Spectral 4’, 6’‐Diamidino‐2‐phenylindole (DAPI) for 5 min before coverslipping with Prolong Gold Anti-fade Mounting Media (Invitrogen). All staining took place at room temperature.

### Image acquisition and analysis

All slides were scanned and imaged using a Vectra Polaris (Akoya Biosciences) multispectral fluorescent slide scanner and analyzed using the HALO image analysis software platform version 3.1 (Indica Labs, Albuquerque, NM). Astrocyte immunoreactivity, measured in terms of mean GFAP fluorescent intensity and percent positive staining area, was assessed in three representative regions of the cortical ribbon in the dorsolateral frontal cortex: at the top of the cortical surface in the subpial glial plate (SGP) [50] or layer 1, at the bottom of the cortex at the interface between the grey and white matter (the grey-white matter interface (GWMI)), and the intervening grey matter spanning between the SGP and the GWMI (cortex).

**Fig. 1:**
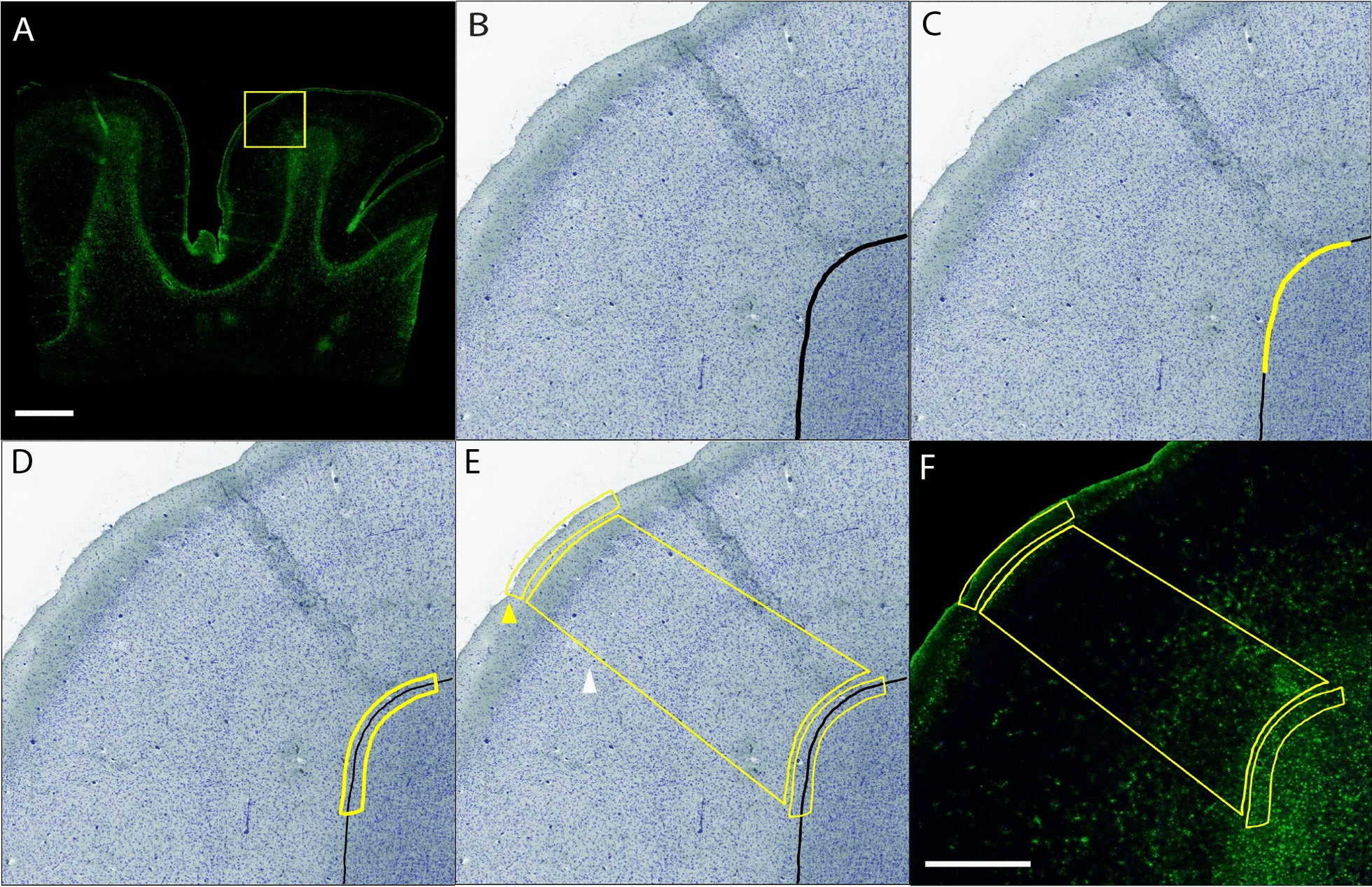
Annotation Regions. The average fluorescent intensity and percent positivity of GFAP were quantified in representative sections of the cortical gyrus (yellow box in **A**, field of view in **B**–**F**) and sulcus (not highlighted) using a modified version of the Area Quantification FL analysis algorithm in HALO. Annotations were generated by switching to the brightfield view and using the magnetic pen tool to delineate the grey-white matter border in the dapi and autofluorescence channels (**B**, black). The pen tool was then used to outline a 2 mm portion of the grey-white border (**C**, yellow), and a 200 µm GWMI annotation was produced using the marginal partitioning tool (**D**, yellow). A 2 mm line was then drawn at the cortical surface and the marginal partitioning tool was used to generate a 200 µm subpial annotation (**E**, yellow arrow head). The brush tool was used to annotate the cortical grey matter spanning between the subpial and GWMI annotations (**E**, white arrow head). Composite image showing all annotations with the GFAP channel in darkfield view (**F**). Green = GFAP, blue = DAPI. Scale bars: **A** = 5 mm, **B**–**F** = 1 mm

The selection of each annotated region of interest is illustrated in Fig. 1. Annotations were generated by switching to the brightfield view and using the magnetic pen tool to delineate the grey-white matter border in the dapi and autofluorescence channels (Fig. 1B). The pen tool was used to outline a 2 mm portion of the grey-white border (Fig. 1C), and a 200 µm GWMI annotation was produced using the marginal partitioning tool (Fig. 1D). A 2 mm line was drawn at the cortical surface and the marginal partitioning tool was used to generate a 200 µm subpial annotation (Fig. 1E, yellow arrowhead). The brush tool was used to annotate the cortical grey matter spanning between the subpial and GWMI annotations (Fig. 1E, white arrowhead). All annotations were generated at the gyral crest and depth of the sulcus on each slide.

Astrogliosis, indicated by level of GFAP immunoreactivity, in each of these representative cortical compartments was quantified using a modified version of the Area Quantification FL analysis settings algorithm in HALO (Indica Labs, v2.1.5). The algorithm was thresholded based on detection of positive pixel staining while minimizing background. All slides were analyzed using the same analysis algorithm and the percent positive area and mean fluorescent intensity of GFAP-positive pixels in each annotation region at the crest and sulcus were recorded for each image. All analyses were conducted blinded to donor identity and diagnosis.

### Statistical analyses

Statistical analyses were run using Graph Pad Prism (version 9). Data were tested for outliers and normality using a Shapiro–Wilk test. Group means reflecting levels of astrogliosis in the different tissue compartments (SGP, Cortex, GWMI) were compared using one-way ANOVAs with Dunnett’s post-hoc tests for normally distributed data, or Kruskal–Wallis with Dunn’s post-hoc tests for non-parametric data. Potential differences in astrogliosis present at the gyrus versus the sulcus were calculated within each cohort using a ratio of sulcus:gyrus GFAP. Ratios were log transformed and compared using one-way t-tests.

## Results

**Fig. 2:**
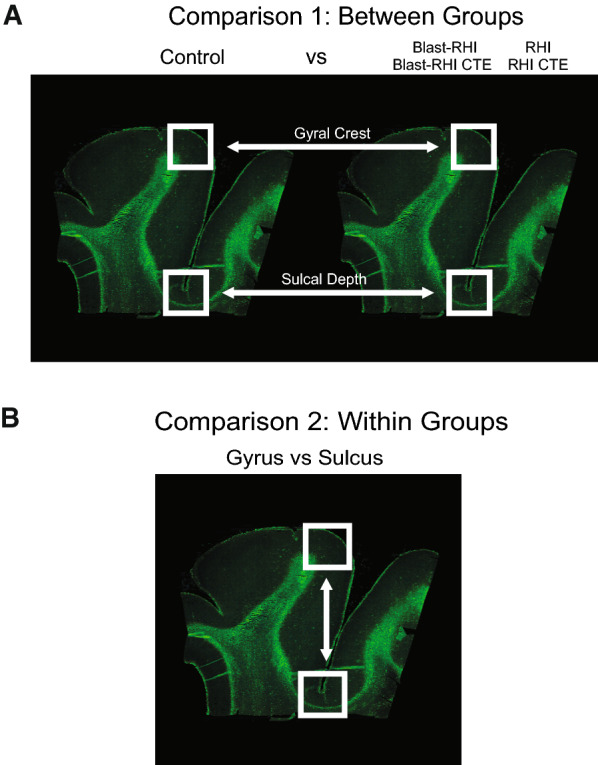
Overview of GFAP Comparisons. Summary of the two main types of comparisons carried out in the current study. Comparison 1 (**A**) directly compares the GFAP immunoreactivity (percent positivity and mean fluorescent intensity) between Controls and each neurotrauma group at both the gyral crest and sulcal depth. Comparison 2 (**B**) directly compares the GFAP at the sulcus to the GFAP at the gyrus within each cohort using a relative ratio of sulcal:gyral GFAP, where a value over 0 indicates sulcal predominance, and a value under 0 indicates gyral predominance

We conducted two sets of comparisons, outlined in Fig. 2. The first type of analysis was a direct comparison of GFAP expression in the three distinct cortical compartments either at the gyral crest or sulcal depth across all cohorts (Fig. 2A, Comparison 1). The second analysis was a direct comparison of GFAP expression at the sulcus versus crest within each group (Fig. 2B, Comparison 2). In this second comparison, the ratio of sulcus:gyrus GFAP was calculated for each donor. Values significantly greater than zero indicated higher GFAP at the sulcus (sulcal predominance), while values significantly lower than zero indicated higher GFAP at the gyrus (gyral predominance).

### Between group comparisons of GFAP at the gyral crest

**Fig. 3:**
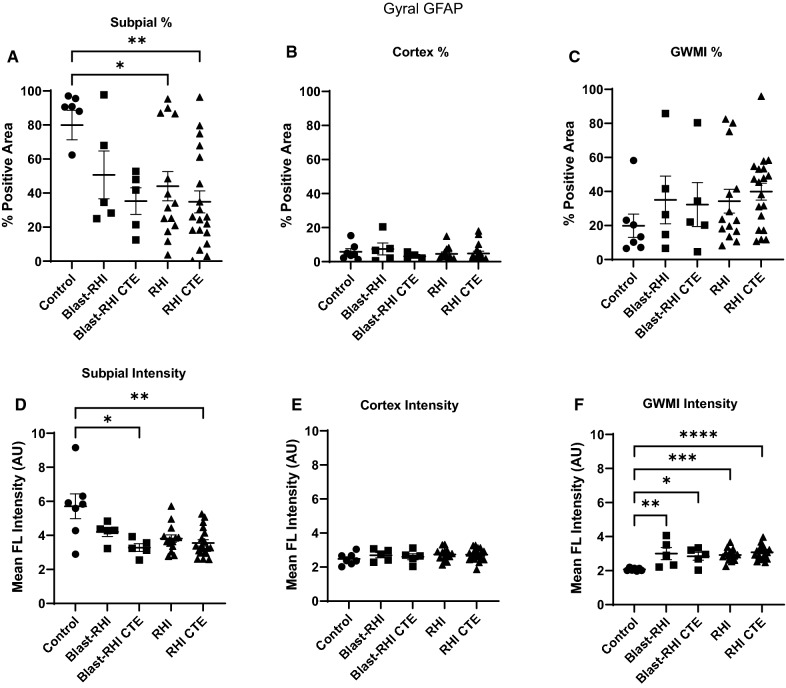
Between Group Comparisons of GFAP at the Gyral Crest. At the crest of the gyrus, GFAP was significantly decreased at the subpial surface in the RHI (**A**) Blast-RHI CTE (**D**), and RHI-CTE (**A**, **D**). No differences were detected in the underlying grey matter (**B**, **E**). At the grey-white matter interface, all neurotrauma groups had significantly higher mean fluorescent GFAP intensity compared to controls (**F**). No significant differences were detected in percent positivity at the GWMI (**C**). Error bars represent standard error of the mean. *P < 0.05, **P < 0.01, ***P < 0.001, ****P < 0.0001

There was a significant decrease in subpial GFAP percent positivity at the gyral crest among the RHI and RHI CTE groups compared to controls (Fig. 3A, RHI vs Control: *p* = 0.0297, RHI CTE vs Control: *p* = 0.0025). The Blast-RHI CTE and RHI CTE groups had significantly lower mean GFAP intensity in this same area (Fig. 3D, Blast-RHI CTE: *p* = 0.0232, RHI CTE: *p* = 0.0096). There were no significant differences detected between groups in GFAP percent positivity or intensity within the grey matter immediately subjacent to the subpial plate (Fig. 3B,E, *p* > 0.05). All neurotrauma cohorts had significantly higher levels of astrogliosis in terms of mean fluorescent GFAP intensity at the grey-white matter interface compared to controls (Fig. 3F, Blast-RHI vs Control: *p* = 0.0019, Blast-RHI CTE vs Control: *p* = 0.0114, RHI vs Control: *p* = 0.0003, RHI CTE vs Control: *p* < 0.0001), though the percent GFAP-positive staining in this region did not reach statistical significance (Fig. 3C, *p* > 0.05).

### Between group comparisons of GFAP at the sulcal depths

**Fig. 4:**
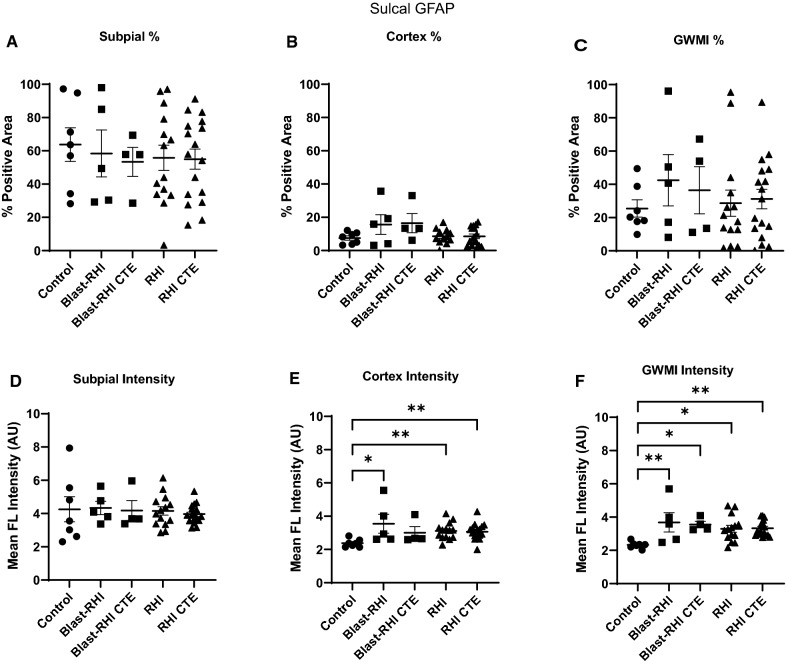
Between Group Comparisons of GFAP at the Sulcal Depth. At the depth of the sulcus, no significant differences in GFAP were detected at the subpial surface (**A**, **D**). Within the underlying cortical grey matter, the Blast-RHI, RHI), and RHI CTE) groups all had significantly higher mean fluorescent GFAP intensity compared to controls (**E**), with no differences detected in any groups for percent positivity (**B**). At the grey-white matter junction, all neurotrauma groups had significantly higher GFAP in terms of mean fluorescent intensity compared to controls (**F**). No differences were detected in percent positivity at the GWMI (**C**). Error bars represent standard error of the mean. *P < 0.05, **P < 0.01

No significant differences in GFAP at the subpial surface were found between groups at the depth of the sulcus in either percent positivity or fluorescent intensity (Fig. 4A, D, *p* > 0.05). However, differences were detected between groups within the subjacent cortex and at the grey-white matter interface. Specifically, the Blast-RHI, RHI, and RHI-CTE groups had significantly higher mean fluorescent GFAP intensity in the sulcal cortex compared to controls (Fig. 4E, Blast-RHI vs Control: *p* = 0.0456, RHI vs Control: *p* = 0.0058, RHI CTE vs Control: *p *= 0.0038), and similar changes were found in the Blast-RHI, Blast-RHI CTE, RHI, and RHI-CTE groups compared to controls at the grey-white matter junction (Fig. 4F, Blast-RHI: *p* = 0.0043, Blast-RHI CTE vs Control: *p* = 0.0187, RHI vs Control: *p* = 0.0106; RHI CTE vs Control: *p* = 0.0059).

### Within group comparisons of gyral versus sulcal GFAP + astrogliosis

**Fig. 5:**
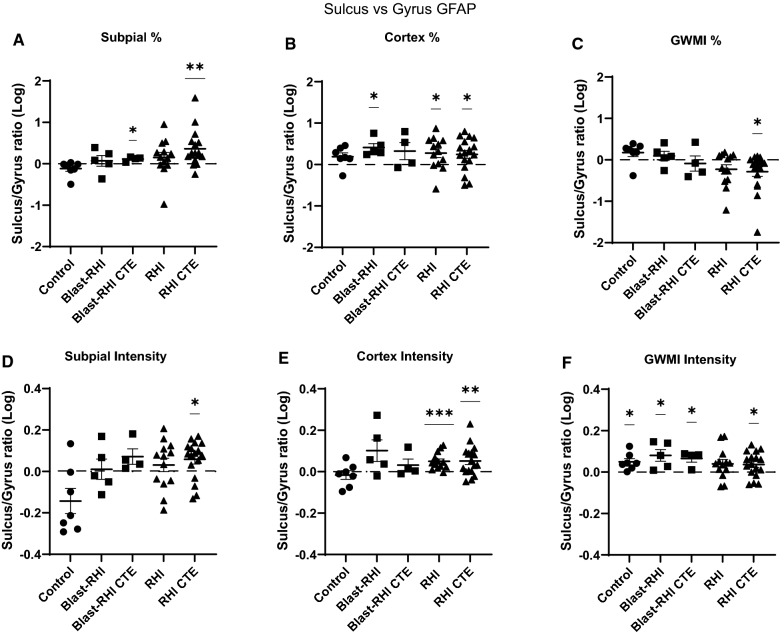
Within Group Comparisons of Sulcal vs Gyral GFAP. Within group comparisons of sulcal versus gyral GFAP were calculated using a relative ratio of sulcal:gyral GFAP. The Blast-RHI CTE group had higher GFAP at the sulcus relative to the gyrus in terms of percent positivity at the subpial surface (**A**), while the RHI-CTE group had higher GFAP at the sulcus relative to the gyrus at the subpial surface in both percent positivity and mean intensity (**A**, **B**). Within the cortex, the Blast-RHI group had significantly higher GFAP in the sulcus in terms of percent positivity (**B**), while the RHI and RHI CTE groups had significantly greater sulcal predominance in terms of both percent positivity (**B**) and mean fluorescent intensity (**E**). At the GWMI (**C**, **F**), all groups except RHI had significantly higher GFAP intensity at the sulcus relative to the gyrus (**F**). The RHI CTE group had significantly less at the sulcus in terms of percent positivity (**C**). Ratio data were log transformed and analyzed using one-way t-tests. Values significantly greater than zero indicated higher GFAP at the sulcus (sulcal predominance), while values significantly lower than zero indicated higher GFAP at the gyrus (gyral predominance). Error bars represent standard error of the mean. *P < 0.05, **P < 0.01, ***P < 0.001

When comparing relative ratios of sulcus:gyrus GFAP within each cohort at the subpial surface (Fig. 5A, D), only the CTE cohorts had significant differences: the Blast-RHI CTE (*p* = 0.0221) and RHI CTE (*p* = 0.0022) groups both had significantly higher percent positivity of GFAP at the sulcus (Fig. 5A), while the RHI CTE (*p* = 0.017) group also had significantly higher mean fluorescent intensity in the same area compared to the gyrus (Fig. 5D). Within the grey matter ribbon spanning between the SGP and the GWMI (Fig. 5B, E), the Blast-RHI, RHI, and RHI CTE groups all had significantly higher levels of GFAP at the sulcus versus crest, though in different ways: Blast-RHI (*p* = 0.0116) had more in the sulcus in terms of percent positivity (Fig. 5B), while RHI and RHI-CTE had more in terms of both percent positivity (Fig. 5B, RHI: *p* = 0.0246, RHI CTE: *p* = 0.0221) and mean fluorescent intensity (Fig. 5E, RHI: *p* = 0.0008, RHI CTE: *p* = 0.0093). Meanwhile, at the GWMI (Fig. 5C, F), the Controls (*p* = 0.018), Blast-RHI (*p* = 0.0458), Blast-RHI CTE (*p* = 0.0368), and RHI CTE (*p* = 0.0241) cohorts had significantly higher mean fluorescent GFAP intensity at the depth of the sulcus compared to the gyral crest (Fig. 5F). Interestingly, the RHI CTE group also had significantly lower GFAP percent positivity at the grey-white matter junction in the sulcus compared to the gyrus (Fig. 5C, *p* = 0.0218).

## Discussion

We found increased GFAP fluorescence intensity indicative of reactive astrogliosis at the grey-white matter boundary in subjects with a history of RHI or blast injury compared to controls without TBI. We also found mixed results, including significantly lower GFAP at the subpial surface at the crest of the gyrus in the same groups compared to controls, but no change at the sulcus. These findings suggest that neurotrauma alone, even in the absence of CTE pathology, might cause astrocytes to alter their GFAP expression [8], and that the alteration occurs in a region-dependent manner.

Astrocytes become reactive in response to varying external stimuli, such as microglia-released cytokines [23, 31] and extravasated serum proteins from a disrupted blood–brain barrier [49]. The pattern of intense astrogliosis at the grey-white matter junction detected here and in previous studies [47, 50] might reflect the localization of shearing forces that occur in this area during blast and impact neurotrauma. Both types of neurotrauma involve diffuse brain injuries that result from the transfer of inertial forces caused by rapid acceleration/deceleration of the head [10, 19, 22]. The injury severity and geometry of the mechanical loading sites are associated with the extent of the diffuse damage [51]. Certain brain areas are more susceptible to injury after neurotrauma than others, such as the interface between the grey and white matter [13, 20, 42]. It is, therefore, predictable that the consequences of blast and RHI would produce similar neuropathological effects, including increased interface astrogliosis.

Our findings support and extend previous studies [22, 26, 27, 30, 36, 48, 50, 54]. In animal models of blast injury, increased GFAP has been reported in the cortex and hippocampus of swine [30], at the grey-white matter interface in ferrets [47], and throughout the cortex and diencephalon in mice [22, 27]. Similar findings have also been reported in animal models of RHI, including robust GFAP-positive astrogliosis in various grey and white matter regions in mice [40, 43, 45, 54] and in the cortex and subcortical white matter in ferrets [48]. Persistent astrogliosis has also been documented in studies of postmortem human brains exposed to blast [50] and impact head trauma [26, 54], and is a known feature of CTE [36]. However, this is the first study to quantitatively evaluate astrogliosis in distinct cortical compartments in blast and RHI-exposed postmortem human tissue. The discrepancy between the fluorescent intensity and percent positive area results reported might reflect a non-proliferative astrogliosis process, whereby GFAP is upregulated, resulting in increased fluorescence, without altered astrocytic density [53]. This finding might also reflect the presence of non-scar forming reactive astrocytes in blast and RHI-related neurotrauma.

We expected all donors with a history of neurotrauma to have elevated astrogliosis compared to controls, as previous studies have shown increases in neuroinflammation, reactive astrogliosis and GFAP expression after head trauma. Furthermore, we expected this increase to be highest in tissue compartments most subject to shearing forces from RHI, such as the grey-white matter junction at the depth of the cortical sulcus. However, not all our results were consistent with these predictions. While the RHI-CTE group did have significantly higher GFAP at the grey-white matter interface in both the sulcus and gyrus compared to controls, when compared within group they also had a lower density of astrogliosis at the sulcal GWMI compared to the gyral GWMI. The loss of GFAP immunoreactivity detected at the gyral subpial surface in the neurotrauma cohorts was also unexpected. It is unclear from the current studies why these discrepant results occurred, as they relied on GFAP as the sole marker of reactive astrocytes.

A recent study looking at genetic alterations in the sulcus versus crest in RHI, low CTE, and controls reported altered glial responses as an innate feature of the sulcus [11]. This study also showed a complex mix of increased and decreased inflammatory processes, including some related to astrocyte development. Some alterations in genes related to immune and inflammation processes were also found to be unique to the sulcus, relative to the other groups. Taken together, these findings suggest a complex interplay of alterations in the gyral crest and sulcus after head trauma. Future studies using additional astrocyte injury markers will be useful for elucidating the post-traumatic astrocytic response in these distinct brain regions.

There are several limitations to this study, including the use of restricted cortical sampling and the small number of cases assessed in the Blast-RHI, Blast-RHI CTE, and Control cohorts due to tissue availability. Additionally, the unique nature and heterogeneous mechanisms of blast injury make quantification of exposure challenging and non-uniform. For instance, blast exposure may involve multiple mechanisms of trauma, including blast wave (primary), acceleration of debris (secondary), body displacement (tertiary), and burns, toxic gases, and crush injuries (quaternary)[10]. While we focused on cases with known exposure to primary blast injury, most of the donors in the blast groups had concomitant RHI exposure that makes it difficult to isolate the specific contribution of blast- vs impact-related injuries to the astrocytic alterations reported here. Nevertheless, donors in the Blast-RHI without CTE cohort had significantly less RHI exposure compared to the other neurotrauma cohorts and still had significantly higher GFAP at the grey-white matter interface compared to controls. This suggests that blast exposure alone, without significant contact sport RHI, might be sufficient to elicit chronic astrocytic alterations.

**Fig. 6:**
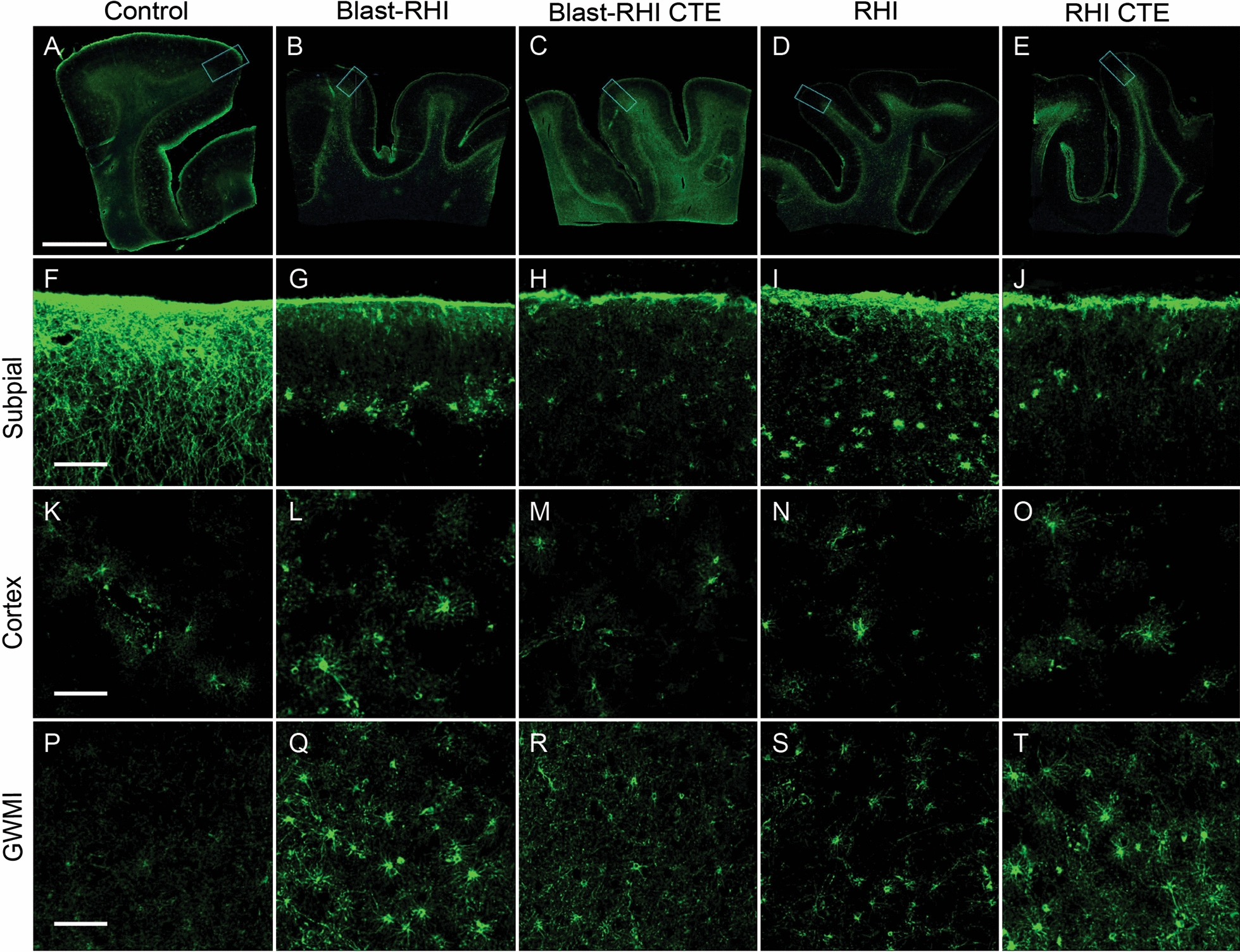
GFAP + Astrocyte Morphology in the Gyral Crest of the Dorsolateral Frontal Cortex. Representative images of the dorsolateral frontal cortex stained with GFAP from Control (**A**), Blast-RHI (**B**), Blast-RHI CTE (**C**), RHI (**D**), and RHI CTE (**E**) cases. The blue rectangular boxes in **A**–**E** indicate the areas analyzed and discussed in F-T. Astrocytes of the glial limitans superficialis demonstrate intralaminar astrocyte processes that extend down from the subpial surface into cortical layers I-II in the control case (**F**). In all forms of neurotrauma (**G**–**J**), there is a dropout of interlaminar astrocytic processes, however astrocytes visible in the subjacent layer display increased GFAP expression and swollen cell bodies indicative of reactive astrocytosis. Throughout the cortex astrocytes appear similar across groups (K-O). At the GWMI, the control case (P) demonstrates fine astrocytic processes and low levels of GFAP expression, while the neurotrauma groups (Q-T) display greater GFAP expression and reactive profiles with thicker astrocyte processes. Green = GFAP. Scale bar, top panel (**A**–**E**): 5 mm. Scale bar, bottom panels (**F**–**T**): 50 μm

The lack of a CTE-specific effect on GFAP expression observed in the Blast-RHI CTE and RHI CTE groups, compared to their non-CTE counterparts, might be due to inclusion of only mild CTE cases (low stage, McKee Stages I-II) that have significantly less p-tau pathology than later stage disease (high stage, McKee Stages III-IV)[5]. In addition, while GFAP fluorescent intensity is commonly used to assess astrogliosis, it is inherently an indirect measurement. Morphological assessment of reactive astrocytes is another commonly used technique [16]. In the present study clear differences were apparent in astrocyte morphology between controls and the neurotrauma cohorts, such as reduced interlaminar astrocytic processes and swollen astrocyte cell bodies at the subpial surface (Fig. 6F-J), as well as increased GFAP, cellular hypertrophy, and thickening of astrocyte processes at the grey-white matter interface (Fig. 6P-T). These features are consistent with reactive astrocyte morphologies [53].

Future studies should incorporate a multipronged approach using a combination of markers and techniques, including quantitative morphological analyses, to fully capture the effect of neurotrauma on regional reactive astrogliosis [16]. Integration of clinical data, including cognitive, behavioral, and mood alterations, with neuropathological outcomes will also be useful to identify the long-term clinical effects of these injuries. Assessment of reactive astrocyte profiles in larger cohorts, with a range of different blast and RHI exposure levels, in conjunction with neuropathological analysis of diverse brain regions, will further elucidate the role of astrocytes in the progression of TBI-induced neurodegenerative changes.

This work has translational significance considering the potential clinical utility of astrocyte-focused biomarkers. Advanced imaging techniques are used to follow individuals exposed to neurotrauma, such as proton magnetic resonance spectroscopy (^1^H-MRS) to detect astrocyte metabolism [24, 32]. A recent in vivo ^1^H-MRS study of retired professional football players found significant correlations between clinical cognitive symptoms and different neurochemicals associated with inflammation, including the neurometabolite myo-inositol (mIns) [2]. Altered mIns has been associated with changes in astrocyte activation state [24, 28], and is a potentially relevant imaging modality for the study of reactive astrocytes in vivo. GFAP released from astrocytes following neurotrauma can be also measured in blood, and correlates with injury severity, suggesting utility as a peripheral biomarker as well [21, 56].

## Conclusions

In summary, although considerable research has been directed towards neuronal and axonal injury and accumulations of neurodegenerative proteins following head trauma, the role of astrocytic alterations in post-traumatic injury processes are just beginning to be recognized. The results presented here quantitatively highlight increased GFAP expression at the cortical grey-white matter interface as a neuropathological hallmark of mild blast or impact neurotrauma.

## Data Availability

The datasets generated and analyzed in the current study are available from the corresponding author upon reasonable request.

## Abbreviations

AD: Alzheimer’s disease
ADC: Alzheimer’s Disease Center
BBB: Blood–brain barrier
CTE: Chronic traumatic encephalopathy
DAPI: 4’, 6’‐Diamidino‐2‐phenylindole
FTLD: Frontotemporal lobar degeneration
GFAP: Glial fibrillary acidic protein
GWMI: Grey-white matter interface
^1^H-MRS: Proton magnetic resonance spectroscopy
HRP: Horseradish peroxidase
IED: Improvised explosive device
IRB: Institutional review board
LBD: Lewy body disease
mIns: Myo-inositol
MND: Motor neuron disease
mTBI: Mild traumatic brain injury
NINDS/NIBIB: National Institute of Neurological Disorders and Stroke/National Institute of Biomedical Imaging and Bioengineering
PBST: Phosphate buffered saline with triton
PLP: Periodate-lysine-paraformaldehyde
PTSD: Post-traumatic stress disorder
RHI: Repetitive head impacts
SGP: Subpial glial plate
TDP-43: Transactive response DNA-binding protein 43
UNITE: Understanding Neurologic Injury and Traumatic Encephalopathy
VA: Veteran’s affairs
VA-BU-CLF: Veteran’s affairs-Boston University-Concussion Legacy Foundation
